# Safety, pharmacodynamic, and pharmacokinetic characterization of vericiguat: results from six phase I studies in healthy subjects

**DOI:** 10.1007/s00228-020-03023-7

**Published:** 2020-10-30

**Authors:** Michael Boettcher, Dirk Thomas, Wolfgang Mueck, Stephanie Loewen, Erich Arens, Kenichi Yoshikawa, Corina Becker

**Affiliations:** 1 Research & Development, Pharmaceuticals, Clinical PD CV, Bayer AG, Wuppertal, Germany; 2 Experimental Medicine, Bayer AG, Wuppertal, Germany; 3 Research & Development, Pharmaceuticals, Clinical PK CV, Bayer AG, Aprather Weg 18a, 42113 Wuppertal, Germany; 4 Chrestos Concept GmbH & Co. KG, Essen, Germany; 5 Present Address: Im Straesschen, Monheim, Germany; 6 Clinical Sciences, Research & Development Japan, Bayer Yakuhin, Ltd, Osaka, Japan

**Keywords:** Heart failure, Pharmacotherapy, Phase I, Pharmacokinetic, Pharmacodynamic

## Abstract

**Purpose:**

To characterize the safety, pharmacodynamics, and pharmacokinetics (PK) of vericiguat in healthy males.

**Methods:**

Six phase I studies were conducted in European, Chinese, and Japanese males. Subjects received oral vericiguat as a single dose (0.5–15.0 mg solution [for first-in-human study] or 1.25–10.0 mg immediate release [IR tablets]) or multiple doses (1.25–10.0 mg IR tablets once daily [QD] or 5.0 mg IR tablets twice daily for 7 consecutive days). Bioavailability and food effects on vericiguat PK (IR tablets) were also studied in European subjects.

**Results:**

Overall, 255 of 265 randomized subjects completed their respective studies. There were no deaths or serious adverse events. Vericiguat was generally well tolerated at doses ≤ 10.0 mg. In the first-in-human study, the most frequent drug-related adverse events were headache and postural dizziness (experienced by five subjects each [7.2%]). Three of four subjects who received vericiguat 15.0 mg (oral solution, fasted) experienced orthostatic reactions. Vericiguat (≤ 10.0 mg, IR tablets) was rapidly absorbed (median time to reach maximum plasma concentration ≤ 2.5 h [fasted]) with a mean half-life of about 22.0 h (range 17.9–27.0 h for single and multiple doses). No evidence for deviation from dose proportionality or unexpected accumulation was observed. Administration of vericiguat 5.0 mg IR tablets with food increased bioavailability by 19% (estimated ratio 119% [90% confidence interval]: 108; 131]), reduced PK variability, and prolonged vericiguat absorption relative to the fasted state.

**Conclusion:**

In general, vericiguat was well tolerated. These results supported further clinical evaluation of vericiguat QD in patients with heart failure.

**Registry numbers:**

EudraCT: 2011-001627-21; EudraCT: 2012-000953-30

**Electronic supplementary material:**

The online version of this article (10.1007/s00228-020-03023-7) contains supplementary material, which is available to authorized users.

## Introduction

Heart failure (HF) is a major healthcare burden [1, 2]. Three subtypes of HF exist: HF with preserved ejection fraction (HFpEF; left ventricular ejection fraction [LVEF] ≥ 50%), HF with mid-range ejection fraction (LVEF 40–49%), and HF with reduced ejection fraction (HFrEF; LVEF < 40%) [3]. Despite the availability of current treatments shown to improve survival in HFrEF [3], around one in six patients with chronic HFrEF will experience a worsening HF event, and this segment of patients is at high risk for mortality and recurrent hospitalization for HF [4].

The pathophysiology of HF involves multiple systems, including the sympathetic nervous system and the renin–angiotensin–aldosterone system [5], which are the targets of current treatment [6]. Nitric oxide–soluble guanylate cyclase–cyclic guanosine monophosphate (NO–sGC–cGMP) signaling contributes to cardiac function [6]. sGCs catalyze cGMP synthesis, leading to tissue relaxation [7]. Impairment of the NO–sGC–cGMP signaling pathway is implicated in cardiovascular, cardiopulmonary, and cardiorenal diseases [6].

In HF, endothelial dysfunction and the presence of reactive oxygen species reduce bioavailability of NO, suppressing the activity of sGC and the production of cGMP [8]. Restoring deficiencies in cGMP through sGC stimulation is a potential approach for the treatment of cardiovascular disease [6–9]. Preclinical studies assessed sGC stimulators and identified vericiguat as a clinical candidate with a suitable pharmacokinetic (PK) profile for once daily (QD) dosing in humans [10].

Vericiguat is a direct stimulator of sGC developed as a first-in-class therapy to reduce the risk of cardiovascular death and hospitalization for HF following a worsening HF event, in adults with symptomatic chronic HF and LVEF < 45%. Vericiguat has been studied in patients with HF with a LVEF < 45% (VICTORIA, NCT02861534 [11, 12] and SOCRATES-REDUCED, NCT01951625 [13]) and in those with a LVEF ≥ 45% (VITALITY-HFpEF, NCT03547583 [4] and SOCRATES-PRESERVED, NCT01951638 [14]).

Here, we describe the results of a basic clinical pharmacology program consisting of six separate phase I studies, including the first study of vericiguat in humans that assessed the safety, tolerability, pharmacodynamics (PD), and PK profile of vericiguat as a single dose (SD) and as multiple doses (MDs) in healthy human volunteers. Collectively, these studies also assessed the influence of the following: ethnicity (Caucasian, Japanese, or Chinese), food (fasted or fed, including high calorie versus standardized breakfast), vericiguat formulation (immediate release [IR] tablet versus solution), and dosing regimen of vericiguat (QD or twice daily [BID]) on the safety, PD, and PK of vericiguat in healthy volunteers.

## Methods

### Study population and study designs

Healthy male subjects aged 18–45 years with a body mass index of 18–30 kg m^−2^ were eligible for inclusion. Key exclusion criteria included the following: a history of severe allergies, non-allergic drug reactions or multiple drug allergies, febrile illness within 1 week before the first study drug administration, and clinically relevant electrocardiographic (ECG) findings. Written informed consent was obtained from individuals in each study.

The safety, tolerability, PD, and PK of vericiguat were investigated in six phase I, randomized, single/double-blind studies conducted between July 2011 and May 2017. The studies included two SD studies (SD1 and SD2), three MD studies (MD1–MD3), and one bioavailability (BA1) study. Individual study designs are shown in Table 1. All studies comprised a screening, treatment, and follow-up period. Randomization was carried out using a computer-generated system. In SD1, at the first dose step, only two subjects per day received study drug with at least 2 h between study drug administrations. Dose escalation proceeded following acceptable safety and tolerability data in the preceding step. Studies met all local legal and regulatory requirements and were conducted in accordance with the currently accepted version of the Declaration of Helsinki, the International Conference on Harmonisation Good Clinical Practice Guideline, the European Union Directive 2001/20/EC, and the German Drug Law (Arzneimittelgesetz).

**Table 1:** Six phase I studies of oral vericiguat in healthy males

Study ID, population, description	Subjects completed of randomized, n	Study design and randomization ratio	Vericiguat dose^a^, fed or fasted, duration	Vericiguat formulation^a^
SD escalation studies				
SD1. European subjects^b^ Safety and tolerability, PK/PD	69 of 73^c^	Randomized, single-blind, parallel group, 8:2 to receive vericiguat or PBO	SD: 0.5, 1.0, 2.5, 5.0, 7.5, 10.0, or 15.0 mg on day 1 (fasted)	PEG solution
SD2. Chinese subjects Safety and tolerability, PK/PD	36 of 36	Randomized, double-blind, 9:3 to receive vericiguat or PBO	SD: 1.25, 5.0, or 10 mg on day 1 (fasted)	1.25 mg IR tablet
MD escalation studies				
MD1. Japanese subjects Combined SD and MD study Safety and tolerability, PK/PD	47 of 48^d^	Randomized, single-center, single-blind, 9:3 to receive vericiguat or PBO per dose step	SD: 1.25, 5.0, 7.5, 10.0 mg on day 1 (fasted) MD: 1.25, 5.0, 7.5, or 10.0 mg QD for 7 days (days 5–11; fasted except for day 5^e^)	1.25 mg IR tablet
MD2. European subjects^f^ Safety and tolerability of SDs and MDs of vericiguat, PK/PD	43 of 46^g^	Randomized, single-center, single-blind, PBO-controlled, group comparison 9:3 to receive vericiguat or PBO, per dose step	MD: 1.25, 5.0, or 10.0 mg QD or 5.0 mg BID (fasted, days 1–7)	1.25 mg IR tablet
MD3. Chinese subjects Combined SD and MD study Safety and tolerability, PK/PD	45 of 46^h^	Randomized, single-center, double-blind, PBO-controlled group comparison 9:3 to receive vericiguat or PBO, per dose step	1.25, 5.0, 10.0 mg QD SD on day 0 (fed, high-fat/high-calorie breakfast) MD over 7 days (days 4–10; fed, standardized breakfast)	1.25 mg or 5.0 mg IR tablet
Bioavailability/food effect study				
BA1. European subjects Safety, tolerability, PK/PD of IR tablets vs oral solution Influence of a high-fat, high-calorie meal on the 5.0 mg IR tablet	15 of 16^i^	Randomized, single-center, open-label, non-PBO-controlled, 4-fold crossover	1.25, 5.0 mg IR tablets (fasted), 5.0 mg oral solution (fasted), 5.0 mg IR tablet (fed) SD on day 0	1.25 and 5.0 mg IR tablets vs 5.0 mg PEG solution

^a^Oral

^b^EudraCT: 2011-001627-21

^c^Four subjects withdrew prior to receiving study drug (one due to elevated C-reactive protein, two at the investigator’s discretion, and one withdrew his consent)

^d^One subject withdrew due to an adverse event of “influenza”

^e^High-fat/high-calorie breakfast

^f^EudraCT: 2012-000953-30

^g^Three randomized subjects withdrew prior to receiving study treatment

^h^One subject discontinued the study prematurely with the reason “withdrawal by subject”

^i^One subject was withdrawn from the study at the investigator’s decision

*BA*, bioavailability; *BID*, twice daily; *ID*, identification; *IR*, immediate release; *MD*, multiple dose; *PBO*, placebo; *PD*, pharmacodynamic; *PEG*, polyethylene glycol; *PK*, pharmacokinetic; *QD*, once daily; *SD*, single dose

### Safety and tolerability assessments

Physical examinations and vital signs, including blood pressure (BP), changes in orthostatic BP, heart rate (HR), ECG parameters, and laboratory examinations of blood and urine samples, were assessed. Adverse events (AEs) and serious AEs (SAEs) were reported from the time that subjects provided written informed consent to study completion. AEs were coded according to the Medical Dictionary for Regulatory Activities (MedDRA) version 15.0–20.0.

### Pharmacodynamic evaluation

PD parameters (e.g., BP, HR, and ECG measures) were also directly related to safety assessments. For hemodynamic profiles, HR measured over 1 min, impedance cardiography (cardiac output, cardiac index [cardiac output/body surface area], and systemic vascular resistance [SVR]), and plasma levels of vasoactive hormones (cGMP, noradrenaline, and adrenaline) were measured.

### Pharmacokinetic evaluation

Blood samples were collected before study drug administration (0 h) and at regular intervals following study drug administration. Vericiguat concentrations in plasma and urine samples were determined using high-performance liquid chromatography with mass spectrometry. The calibration range was from 0.2 μg/L (lower limit of quantification [LLOQ]) to 200 μg/L.

The PK parameters assessed are listed in Supplementary Table 1.

### Statistical analyses

For PD assessments, exploratory comparisons between vericiguat and placebo were performed with analysis of variance (ANOVA) and analysis of covariance, comparing differences between pre-treatment day, first day of dosing, and last day of multiple dosing (where applicable) up to 4 h post-baseline.

The concentrations versus time courses of all analytes were tabulated by treatment. The geometric mean, geometric standard deviation (retransformed standard deviation of the logarithms), coefficient of variation, arithmetic mean, standard deviation, minimum, median, maximum value, and the number of measurements were calculated for each sampling point. Dose proportionality was assessed by an ANOVA (including the factor “treatment”) on log-transformed values of PK characteristics of vericiguat.

## Results

### Baseline characteristics and demographics

A total of 265 subjects were randomized across the six studies, and 255 subjects completed their respective studies (Table 1). Across the studies, baseline characteristics were generally similar (Supplementary Table 2), with the exception of increased weight in European subjects (SD1, MD2, and BA1) compared with Chinese (SD2 and MD2) and Japanese subjects (MD1). Subjects had a mean age range of 27.1–38.5 years and a body mass index range of 21.2–25.2 kg m^−2^. Within study treatment groups of the individual studies, characteristics were similar, except for a lower mean body weight (~ 10.0 kg) in the vericiguat 1.25 mg group relative to other treatment groups in MD2.

Results from the first-in-human study (SD1; Supplementary Fig. 1) are presented here as representative SD data from SD2, and the SD parts of MD1 and MD3. Likewise, results from MD1 are presented here as representative MD data from combined SD and MD studies (MD1–3). Results from the BA1 study present the influence of a high-fat, high-calorie meal on the bioavailability of vericiguat 5.0 mg (tablet).

### Safety, PD, and PK following SD administration of vericiguat 0.5–15.0 mg

#### Safety assessments

There were no deaths or SAEs. In the SD1 study, four subjects were withdrawn before they received study drug: two at the investigator’s discretion, one due to elevated C-reactive protein, and one withdrew his consent. The incidence of AEs was 14.3–100.0% with vericiguat (0.5–10.0 mg) and 23.1% with placebo (Table 2). Of the 69 subjects who received placebo or vericiguat, 30 (43.5%) experienced ≥ 1 treatment-emergent AE (TEAE). The incidence of TEAEs by preferred terms is presented in Supplementary Table 3.

**Table 2:** Overall summary of number of subjects with TEAEs: SD1

*n* (%)	Placebo (*n* = 13)	Vericiguat dose (oral PEG solution)							
		0.5 mg (*n* = 7)	1.0 mg (*n* = 8)	2.5 mg (*n* = 8)	5.0 mg non-smokers (*n* = 7)	5.0 mg smokers (*n* = 6)	7.5 mg (*n* = 8)	10.0 mg (*n* = 8)	15.0 mg (*n* = 4)
Any AE	3 (23.1)	1 (14.3)	2 (25.0)	3 (37.5)	5 (71.4)	3 (50.0)	4 (50.0)	5 (62.5)	4 (100.0)
Any study drug-related AE	1 (7.7)	0	1 (12.5)	3 (37.5)	3 (42.9)	1 (16.7)	4 (50.0)	4 (50.0)	4 (100.0)
Any AE related to procedures	1 (7.7)	1 (14.3)	0 (0.0)	2 (25.0)	1 (14.3)	0	0	2 (25.0)	3 (75.0)
Maximum intensity									
Mild	3 (23.1)	1 (14.3)	2 (25.0)	3 (37.5)	5 (71.4)	3 (50.0)	4 (50.0)	5 (62.5)	3 (75.0)
Moderate	0	0	0	0	0	0	0	0	1 (25.0)
Severe	0	0	0	0	0	0	0	0	0
Maximum intensity for study drug-related AEs									
Mild	1 (7.7)	0	1 (12.5)	3 (37.5)	3 (42.9)	1 (16.7)	4 (50.0)	4 (50.0)	3 (75.0)
Moderate	0	0	0	0	0	0	0	0	1 (25.0)
Severe	0	0	0	0	0	0	0	0	0
AE-related deaths	0	0	0	0	0	0	0	0	0
Any SAE	0	0	0	0	0	0	0	0	0
Discontinuation of study drug due to AEs	0	0	0	0	0	0	0	0	0

*AE*, adverse event; *PEG*, polyethylene glycol; *SAE*, serious adverse event; *SD1*, single-dose study 1; *TEAE*, treatment-emergent adverse event

Drug-related TEAEs were experienced by 21 (30.4%) subjects and were mostly classified under the nervous system organ class (eight [11.6%]) or gastrointestinal disorders (seven [10.1%]). The most frequently reported drug-related TEAEs were headache and postural dizziness (both 7.2%), none of which was reported in subjects treated with placebo. All four subjects who received the highest dose of vericiguat (15.0 mg) experienced ≥ 1 drug-related TEAE and three experienced orthostatic reactions. Therefore, dose escalation was stopped at 15.0 mg. Most TEAEs were mild in intensity, except for three moderate drug-related TEAEs in one subject: sinus bradycardia, orthostatic hypotension, and syncope during the standing BP procedure, approximately 2 h after vericiguat treatment. All TEAEs resolved by the end of study, and there were no clinically relevant drug-related changes in laboratory parameters. Once daily dosing of vericiguat in other phase I studies with Chinese and Japanese subjects (SD2, MD1, and MD3) demonstrated a similar safety profile to that of SD1.

#### Pharmacodynamic assessments

Evaluation of the changes from baseline up to 4 h after drug administration demonstrated increases in HR of 4–10 beats per minute (bpm) in subjects receiving vericiguat 5.0–15.0 mg in SD1. In general, subjects treated with vericiguat demonstrated increases in cardiac output and cardiac index relative to those receiving placebo. Analyses of SD1 showed these increases were evident at vericiguat 5.0 mg or higher. Decreases from baseline in SVR were observed in vericiguat 5.0–15.0 mg dose groups compared with the placebo group. Effects on BP were less consistent and not seen in a dose-dependent manner. Mostly, slight decreases were observed in systolic BP and in diastolic BP (decreases in the range of 2–3 mmHg), associated with the above discussed increases in HR.

**Fig. 1:**
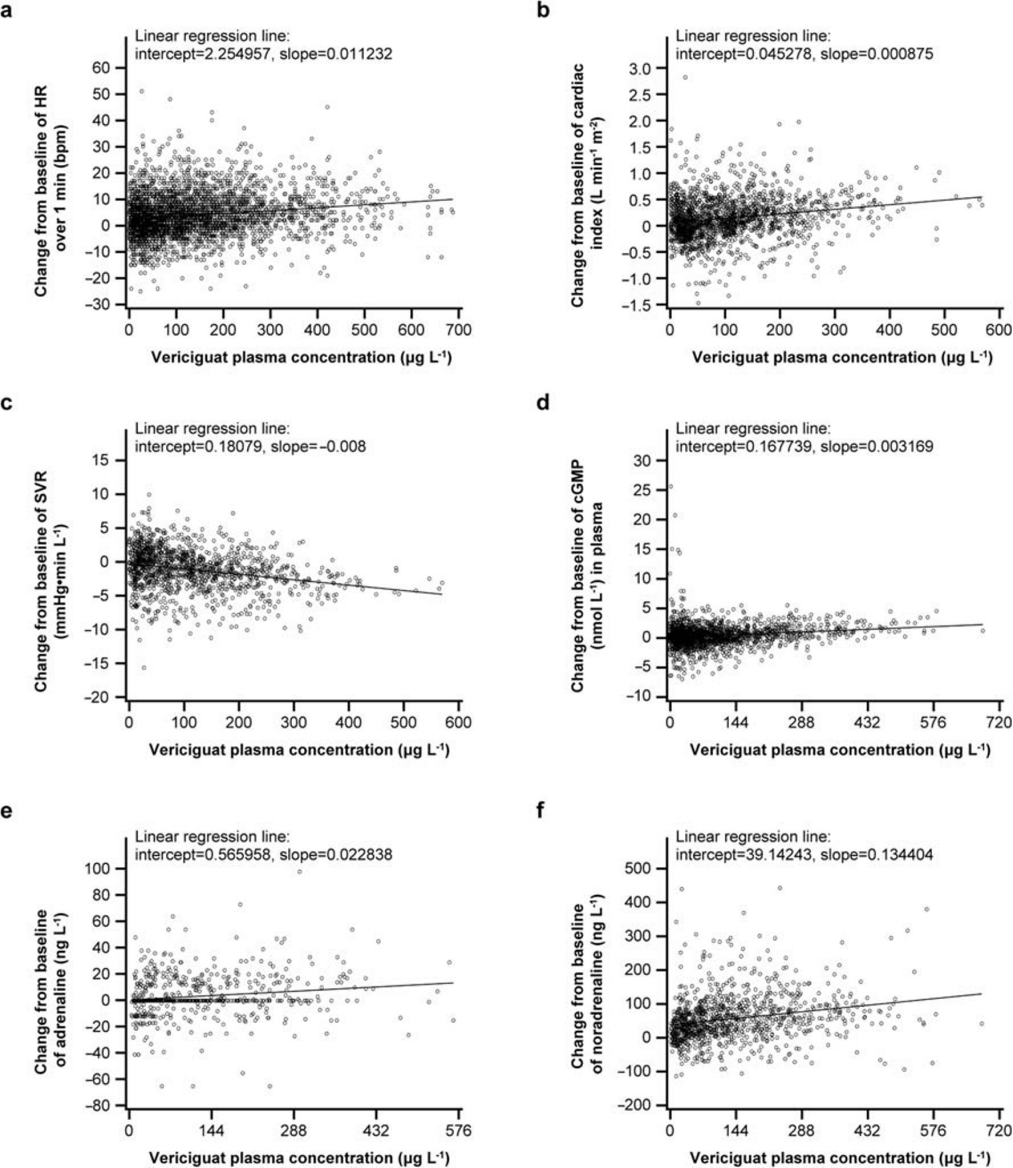
Relationship between vericiguat PK and **a** HR over 1 min, **b** cardiac index, **c** SVR, **d** cGMP, **e** adrenaline, and **f** noradrenaline. Figures include data from: SD1–2, MD1–3, BA1 (HR over 1 min); SD1, MD2, BA1 (cardiac index and SVR); SD1, MD1–2, BA1 (cGMP); SD1, MD1–2 (adrenaline and noradrenaline). *BA*, bioavailability; *bpm*, beats per minute; *cGMP*, cyclic guanosine monophosphate; *HR*, heart rate; *MD*, multiple dose study; *PK*, pharmacokinetic; *SD*, single dose study; *SVR*, systemic vascular resistance

Changes in vasoactive hormones were observed for cGMP, noradrenaline, and plasma renin activity with vericiguat. Immediate and direct correlations between vericiguat plasma concentration and these vasoactive hormones were observed. However, there were no clear dose-dependent relationships. No changes in serum aldosterone were observed, and no statistical tests were performed for angiotensin II and adrenaline, as the values were < LLOQ. Vericiguat plasma concentrations against PD markers for SDs of vericiguat are shown in Fig. 1.

Limited but consistent decreases in creatinine, urea, and uric acid started at either the vericiguat 2.5 mg or 5.0 mg dose steps. cGMP in urine and serum electrolytes did not demonstrate any clear relationship to vericiguat dose administered (data not shown).

#### Pharmacokinetic assessments

**Fig. 2:**
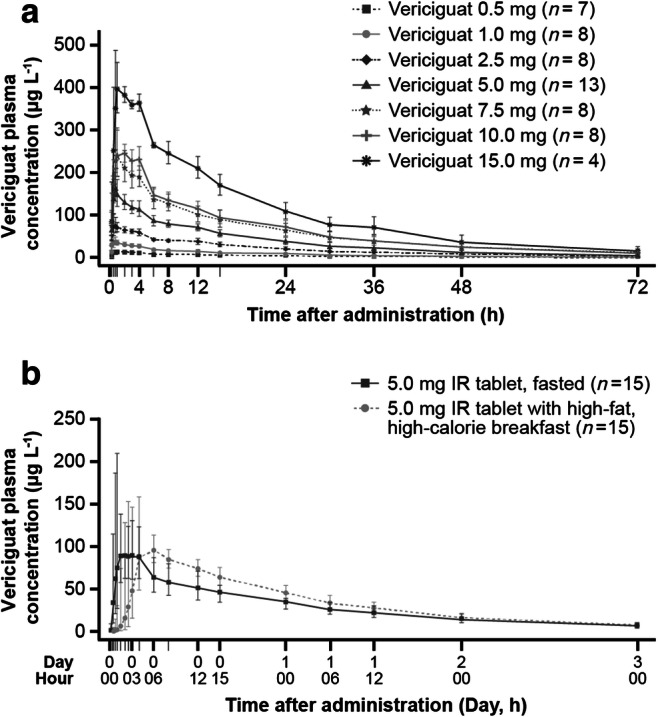
Mean vericiguat plasma concentrations following single oral administration of **a** vericiguat 0.5–15.0 mg as an oral solution in the fasted state (SD1), **b** vericiguat 5.0 mg as an IR tablet in the fasted or fed states (BA1). *BA1*, bioavailability study 1; *IR*, immediate release; *SD1*, single dose study 1

Following single oral doses, vericiguat (0.5–15.0 mg; polyethylene glycol [PEG] solution) was rapidly absorbed, with maximum plasma concentrations reached at a median of between 0.7 h and 1.8 h post-dose. Geometric mean t_1/2_ was 14.5–20.7 h and geometric mean C_max_ increased with dose ranging from 17.2–430.0 μg L^−1^ (Fig. 2a; Table 3). In general, for SDs, the interindividual variability in exposure was low (20–30%). Exploratory testing for dose proportionality in SD1 demonstrated close to linear PK for AUC and a slight trend towards decreasing C_max_ with increasing doses.

**Table 3:** Pharmacokinetic parameters of vericiguat in plasma following single oral doses of vericiguat 0.5–15.0 mg in PEG solution, in the fasted state (day 1, study SD1)

Parameter, unit	Vericiguat dose															
	0.5 mg (*n* = 7)		1.0 mg (*n* = 8)		2.5 mg (*n* = 8)		5.0 mg non-smokers (*n* = 7)		5.0 mg smokers (*n* = 6)		7.5 mg (*n* = 8)		10.0 mg (*n* = 8)		15.0 mg (*n* = 4)	
	Geometric mean (range)	CV, %	Geometric mean (range)	CV, %	Geometric mean (range)	CV, %	Geometric mean (range)	CV, %	Geometric mean (range)	CV, %	Geometric mean (range)	CV, %	Geometric mean (range)	CV, %	Geometric mean (range)	CV, %
C_max_, μg L^−1^	17 (13–22)	20.8	39 (29–54)	23.2	83 (58–112)	23.3	158 (130–192)	12.8	194 (151–228)	15.1	259 (188–364)	20.5	285 (248–315)	8.9	430 (396–491)	9.3
AUC, μg h L^−1^	273 (199–442)	26.9	618 (442–800)	20.1	1450 (1190–1800)	13.9	2900 (2290–4610)	24.1	2500 (2020–3030)	16.3	4460 (3420–6200)	21.9	4940 (3550–6390)	20.0	7900 (6750–9520)	16.2
t_max_^a^, h	1.00 (0.50–2.50)	–	0.75 (0.50–2.50)	–	0.87 (0.48–3.00)	–	1.00 (0.48–2.58)	–	0.73 (0.48–1.00)	–	1.00 (0.73–1.48)	–	1.25 (0.75–4.02)	–	1.75 (0.73–2.50)	–
t_1/2_, h	17.2 (13.9–21.7)	19.8	19.4 (15.9–23.4)	13.9	19.4 (16.2–26.0)	16.9	19.8 (14.8–29.9)	24.4	14.5 (9.7–18.5)	25.9	19.8 (15.1–24.2)	15.9	20.7 (11.5–39.9)	43.9	17.2 (13.8–25.5)	27.8

^a^Median (range)

*AUC*, area under the plasma concentration versus time curve from zero to infinity after single (first) dose; *C*_*max*_, maximum drug concentration in plasma after single dose administration; *CV*, coefficient of variation; *PEG*, polyethylene glycol; *SD1*, single-dose study 1; *t*_*max*_, time to reach maximum drug concentration in plasma after single (first) dose; *t*_*1/2*_, half-life associated with the terminal slope

Mean urinary recovery of unchanged vericiguat was 6–8% (0–72 h after dosing), and excretion of vericiguat occurred mainly during the first 48 h after administration. Geometric mean renal clearance ranged from 0.10 to 0.15 L h^−1^. PK parameters in the SD2 study and SD phases of combined SD and MD studies were in line with those observed in the SD1 study.

The PK parameters following a SD of vericiguat in IR tablet formulation are shown in Supplementary Table 4 (MD1 study). Geometric mean t_1/2_ for IR tablets (SDs) ranged between 18.0 h and 22.0 h. Based on similar bioavailability of the 1.25 mg IR tablet and 5.0 mg PEG solution, demonstrated by dose-normalized exposure (BA1 results) and in line with this dose-comparison, mean C_max_ was higher when vericiguat was administered in PEG solution than as an IR tablet (C_max_ 158 μg L^−1^ [vericiguat 5.0 mg PEG solution] vs 62 μg L^−1^ [vericiguat 1.25 mg IR tablet]). Median t_max_ was similar at 1.0 h for both vericiguat 5.0 mg (PEG solution) and vericiguat 1.25 mg (IR tablet).

### Safety, PD, and PK following MD administration of vericiguat

#### Safety assessments

In the multiple dose phase of MD1, the incidence of TEAEs was 33.3–66.7% with vericiguat (1.25–10.0 mg) and 50.0% with placebo. Drug-related TEAEs were reported in three of nine subjects (33.3%) in each vericiguat group and in four of 12 subjects (33.3%) in the placebo group. The most frequent TEAE was “proteins present in urine” (12.5%), and the most frequent drug-related TEAE was “increased alanine aminotransferase” (6.3%). No trend within treatment groups or vericiguat dose was observed.

Most TEAEs were mild in intensity and there were no TEAEs of severe intensity. Five subjects experienced moderate TEAEs: influenza (one subject in the 5.0 mg group who was treated with oseltamivir and paracetamol but discontinued the study on day 5), nasopharyngitis (two subjects, 5.0 mg), and orthostatic hypotension (two subjects, 10 mg and placebo). All TEAEs resolved by the end of the study and there were no deaths or SAEs.

#### Pharmacodynamic assessments

Vericiguat (1.25–10.0 mg) at steady state had a statistically significant increase in change from baseline in HR over 1 min, up to 4 h post-dose, relative to placebo (Table 4). A placebo-adjusted increase in HR of < 10 bpm was evident with vericiguat 1.25–10.0 mg, which, in general, increased with escalating doses. Despite showing statistical significance, these changes in HR and HR over 1 min were deemed not clinically relevant by the investigator; similarly, there were no apparent clinically relevant changes in BP following administration of vericiguat relative to placebo.

**Table 4:** Pharmacodynamics following multiple oral doses of vericiguat as IR tablets: change from baseline of difference in heart rate over 1 min (day 11 and day − 1), during the 4 h after administration vs placebo (study MD1)

Change from baseline of difference in heart rate over 1 min	Vericiguat dose (IR tablets)			
	1.25 mg	5.0 mg	7.5 mg	10.0 mg
Difference: vericiguat vs placebo (bpm)	2.95	3.75	3.75	5.42
95% CI	0.06–5.85	0.41–7.08	0.85–6.65	2.42–8.42
*p* value	0.046*	0.029*	0.013*	0.001*

*p value < 0.05 *bpm*, beats per minute; *CI*, confidence interval; *IR*, immediate release; *MD1*, multiple dose study 1

From day − 1 to day 11, changes in vasoactive hormones (in MD1) were seen for cGMP, plasma renin activity, and norepinephrine, generally with doses of vericiguat 5 mg and above.

Cardiac impedance measurements (in MD2) showed a decrease in SVR (2.7–3.8 mmHg min/L) on day 7 with vericiguat compared with placebo, together with an increase of cardiac output (0.55–0.72 mL/min) and cardiac index (0.27–0.37 L/min/m^2^) up to the vericiguat 10 mg dose level.

#### Pharmacokinetic assessments

After MDs, vericiguat was rapidly absorbed, with median t_max_ achieved by 2.5 h in MD1. Slight accumulation in area under the concentration versus time curve 0 to 24 h after administration (AUC_[0–24]_) and C_max_ was observed (R_A_AUC: 1.40–1.66 and R_A_C_max_ 1.16–1.44, respectively) for QD; however, no unexpected accumulation was observed. Geometric mean t_1/2_ was in the range 20.7–27.0 h post-dose for MD1. Excretion of unchanged vericiguat in urine accounted for approximately 5–6% of the dose, and renal clearance over 7 days was approximately 60 mL h^−1^ (day 11; Table 5).

**Table 5:** Pharmacokinetic parameters for vericiguat following multiple administrations of vericiguat 1.25–10 mg QD as IR tablets, in the fasted state (day 11; study MD1)

Parameter	Vericiguat dose (IR tablets)							
	1.25 mg (*n* = 9)		5.0 mg (*n* = 8)^a^		7.5 mg (*n* = 9)		10.0 mg (*n* = 9)	
	Geometric mean (range)	CV, %	Geometric mean (range)	CV, %	Geometric mean (range)	CV, %	Geometric mean (range)	CV, %
C_max,ss_, μg L^−1^	89 (69.3–132)	18.6	289 (214–391)	25.1	407 (296–582)	24.2	472 (303–726)	30.6
C_max,ss_,_norm_, kg L^−1^	4.54 (3.63–5.64)	16.7	3.19 (2.31–4.27)	20.0	3.57 (2.87–4.41)	14.5	3.02 (1.79–4.11)	27.5
AUC_τ,ss_, μg h L^−1^	1170 (978–1570)	14.5	3670 (2660–5130)	23.4	4810 (2790–6460)	27.6	6170 (4160–9790)	29.9
AUC_τ,ss,norm_, kg h L^−1^	59.4 (44.2–73.6)	14.7	40.5 (31.3–53.2)	19.8	42.2 (26.8–48.9)	20.2	39.6 (24.5–55.5)	28.2
t_max_^b^, h	1.00 (0.75–2.50)	–	1.75 (0.75–4.00)	–	2.50 (0.75–2.50)	–	2.50 (0.75–2.50)	–
t_1/2_, h	27.0 (17.6–37.4)	23.6	23.5 (15.8–34.4)	30.6	22.1 (17.5–27.3)	15.9	20.7 (16.3–31.4)	25.2
R_A_AUC	1.66 (1.32–2.09)	13.3	1.44 (1.23–1.78)	13.1	1.49 (1.22–2.14)	19.1	1.40 (0.92–2.49)	27.2
R_A_C_max_	1.44 (0.98–2.65)	29.5	1.16 (0.90–1.42)	13.8	1.26 (0.94–2.02)	23.7	1.29 (0.86–2.40)	27.2
R_LIN_	0.98 (0.88–1.13)	8.47	0.89 (0.75–1.14)	13.0	0.94 (0.72–1.28)	20.0	0.83 (0.63–1.41)	22.9
A_E,ur(0–24)_^c,d^, %	6.04 (2.40–9.35)	2.02^¶^	5.18 (3.43–7.36)	1.53^e^	4.75 (2.41–6.71)	1.24^e^	5.01 (2.16–8.38)	1.99^e^
CL_R_^§^, L h^−1^	0.06 (0.03–0.09)	36.6^¶^	0.07 (0.04–0.10)	31.8^e^	0.07 (0.06–0.09)	12.7^e^	0.08 (0.05–0.11)	22.4^e^
CL_ss_/f, L h^−1^	1.07 (0.80–1.28)	14.5	1.36 (0.97–1.88)	23.4	1.56 (1.16–2.69)	27.6	1.62 (1.02–2.41)	29.9

^a^One subject withdrew after day 5

^b^Median (range)

^c^*n* = 8 for A_E,ur(0–24)_ and CL_R_ at doses of 1.25 and 7.5 mg

^d^Values are arithmetic mean

^e^Values are standard deviation

*A*_*E,ur(0–24)*_, rate of amount of drug excreted into urine from 0 to 24 h after administration to the administered dose; *AUC*, area under the plasma concentration versus time curve from zero to infinity after single (first) dose; *AUC*_*τ,ss*_, AUC during any dosing interval at steady state; *AUC*_*τ,ss,norm*_, AUC_τ,ss_ divided by dose (mg) per kg body weight; *CL*_*ss*_*/f*, total body clearance of drug from plasma calculated after oral administration (apparent oral clearance) after steady state; *C*_*max,ss*_, maximum drug concentration in plasma at steady state during a dosage interval; *C*_*max,ss,norm*_, maximum drug concentration in plasma at steady state during a dosage interval divided by dose (mg) per kg body weight; *CV*, coefficient of variation; *IR*, immediate release; *MD1*, multiple-dose study 1; *QD*, once daily; *R*_*A*_*AUC*, accumulation ratio calculated from AUCτ after multiple dosing and AUCτ after single dosing; *R*_*A*_*C*_*max*_, accumulation ratio calculated from C_max_ after multiple dosing and C_max_ after single dosing; *R*_*LIN*_, linearity factor of pharmacokinetics after multiple administration of identical doses calculated from AUCτ after multiple dosing and AUC after single dosing; *t*_*max*_, time to reach maximum drug concentration in plasma after single (first) dose; *t*_*1/2*_, half-life associated with the terminal slope

Although dose proportionality could not be concluded based on the ANOVA results of treatment ratios of vericiguat 1.25/10.0 mg in MD1, exploratory analyses in MD2 and MD3 indicated no deviation from dose proportionality. Furthermore, steady-state conditions of vericiguat plasma concentrations were reached after approximately 48–72 h post-dose. Higher accumulation rates were observed with the 5.0 mg BID regimen than with the QD regimens in MD2 (R_A_AUC: 2.73; R_A_C_max_: 2.29).

### Bioavailability study

For the 5.0 mg IR tablet, intake of the tablet together with a high-fat, high-calorie breakfast led to a delay of absorption (Fig. 2b), with a median t_max_ of 4 h compared with 1.0–1.5 h in the fasted state. AUC and C_max_ were slightly higher in the fed state, by 19% (estimated ratio [90% CI]: 119% [108; 131]) and 9% (estimated ratio [90% CI]: 109% [92; 129]), respectively, and were less variable with a narrower coefficient of variation range than in the fasted state [15].

The relative bioavailability of the 5 mg tablet versus the 5 mg oral solution administered in the fasted state was reduced by 29% (estimated ratio [%] and 90% CI: 71% [64; 78]), and mean C_max_ by 40% (estimated ratio [90% CI]: 60% [51; 71]). Drug elimination was not affected by formulation, dose, or intake of the IR tablet with food as demonstrated by similar t_1/2_ (approximately 20 h) after all administrations.

Overall, fewer subjects experienced study drug-related TEAEs when vericiguat was administered in the fed state than in the fasted stated (6.3% vs 6.7–25.0%).

## Discussion

These six separate phase I clinical pharmacology studies assessed the safety, PD, and PK profiles of vericiguat in healthy young men.

Vericiguat, at doses of up to 10.0 mg QD for 7 days, was generally well tolerated in European, Chinese, and Japanese healthy men. In SD1, treatment with vericiguat 15.0 mg as an oral PEG solution was not well tolerated due to orthostatic reactions, most likely deriving from the mechanism of action of vericiguat. Therefore, no additional dose escalations were performed. Drug-related TEAEs were mostly nervous system disorders, such as headache and postural dizziness, which could be associated with the mode of action of vericiguat (i.e., vasodilation), or gastrointestinal disorders that could be associated with either the effect of vericiguat on smooth muscle cells (i.e., relaxation), or the intake of PEG, such as diarrhea, nausea, and abdominal discomfort. Therefore, the 15.0-mg dose in PEG solution formulation was not further evaluated in this clinical program.

The observed safety and PD effects of vericiguat are consistent with the mode of action of a sGC stimulator [6]; i.e., relaxation of the smooth muscle in the vasculature leading to changes in hemodynamics [2]. In accordance with the established pharmacological profile of vericiguat in pre-clinical experiments [10], the expected hemodynamic effects were observed in healthy subjects. Specifically, an increase in heart rate was observed as a compensatory reaction to the blood pressure-lowering activity of vericiguat through the baroreflex.

Here, hypotension/orthostatic hypotension was observed in three studies (SD1, MD1, and MD3). Syncope was observed in SD1 only, in which vericiguat was administered in the fasted state and in PEG solution formulation, which was not used in later development.

Mild increases in placebo-adjusted HR (up to 6 bpm), changes in cardiac impedance parameters, and increases in vasoactive hormones were observed with vericiguat at doses of 5.0 mg and above. These changes were consistent with the pharmacological mode of action of vericiguat and corresponded with the expected vasodilation and compensatory increases in HR.

Based on the results of these studies in healthy volunteers, a SBP-guided titration regimen was first examined in the SOCRATES REDUCED study [13] and subsequently implemented in VICTORIA [11, 12]. The starting dose, titration, and the titration interval duration of 2 weeks were selected based on the observed direct relationship between vericiguat plasma concentrations and hemodynamic effects in healthy subjects following ad hoc dosing of different doses of vericiguat and multiple dose administration. For SDs, the PK results demonstrated that vericiguat (PEG solution) in the fasted state was rapidly absorbed (median t_max_ up to 1.75 h), with low interindividual variability in exposure. Mean urinary recovery was in the range of 6–8%, indicating that renal excretion of vericiguat is driven solely by passive filtration. Across the dose range of vericiguat evaluated (0.5–15.0 mg), exploratory testing of dose proportionality using standard bioavailability/bioequivalence criteria (CI 0.8–1.25) [16, 17] demonstrated dose proportionality of PK for AUC and slightly less than dose-proportional increases in C_max_ with increasing doses. Geometric mean t_1/2_ for IR tablets ranged 18.0–22.0 h, supportive of QD dosing.

Exposure and C_max_ following administration of MDs of vericiguat were similar to those following a SD, which indicated time-independent PK.

Increased bioavailability and reduced variability observed in the fed state relative to the fasted state supported administration of vericiguat with food. Drug elimination was not affected by formulation, dose, or administration with food or ethnicity.

In summary, these results were consistent with those previously published for sGC stimulators [18] and in patients with HF [13, 14, 19]. In conclusion, vericiguat QD up to 10.0 mg was generally well tolerated by healthy European, Chinese, and Japanese subjects. Changes in PD measures indicated significant vasodilatory effects at vericiguat doses of 5.0 mg and above. PK parameters were supportive of vericiguat QD dosing in the fed state. The results supported the further evaluation of vericiguat 1.25–10.0 mg in phase II studies [13, 14] and 2.5–10.0 mg in the phase III VICTORIA study [11] as well as the selection of the titration dosing regimen.

## Electronic supplementary material

ESM 1 (PNG 64 kb)

High Resolution Image (TIF 371 kb)

ESM 2 (DOCX 76 kb)

## Data Availability

Availability of the data underlying this publication will be determined according to Bayer’s commitment to the EFPIA/PhRMA “Principles for responsible clinical trial data sharing”. This pertains to scope, timepoint, and process of data access. As such, Bayer commits to sharing upon request from qualified scientific and medical researchers patient-level clinical trial data, study-level clinical trial data, and protocols from clinical trials in patients for medicines and indications approved in the United States (US) and European Union (EU) as necessary for conducting legitimate research. This applies to data on new medicines and indications that have been approved by the EU and US regulatory agencies on or after January 1, 2014. Interested researchers can use http://www.clinicalstudydatarequest.com to request access to anonymized patient-level data and supporting documents from clinical studies to conduct further research that can help advance medical science or improve patient care. Information on the Bayer criteria for listing studies and other relevant information is provided in the Study sponsors section of the portal. Data access will be granted to anonymized patient-level data, protocols, and clinical study reports after approval by an independent scientific review panel. Bayer is not involved in the decisions made by the independent review panel. Bayer will take all necessary measures to ensure that patient privacy is safeguarded.
