# Supplementary figures and images for: Safety, pharmacodynamic, and pharmacokinetic characterization of vericiguat: results from six phase I studies in healthy subjects
Source: Eur J Clin Pharmacol. 2020 Oct 30;77(4):527–37. doi: 10.1007/s00228-020-03023-7 (PMC7935833; doi:10.1007/s00228-020-03023-7)

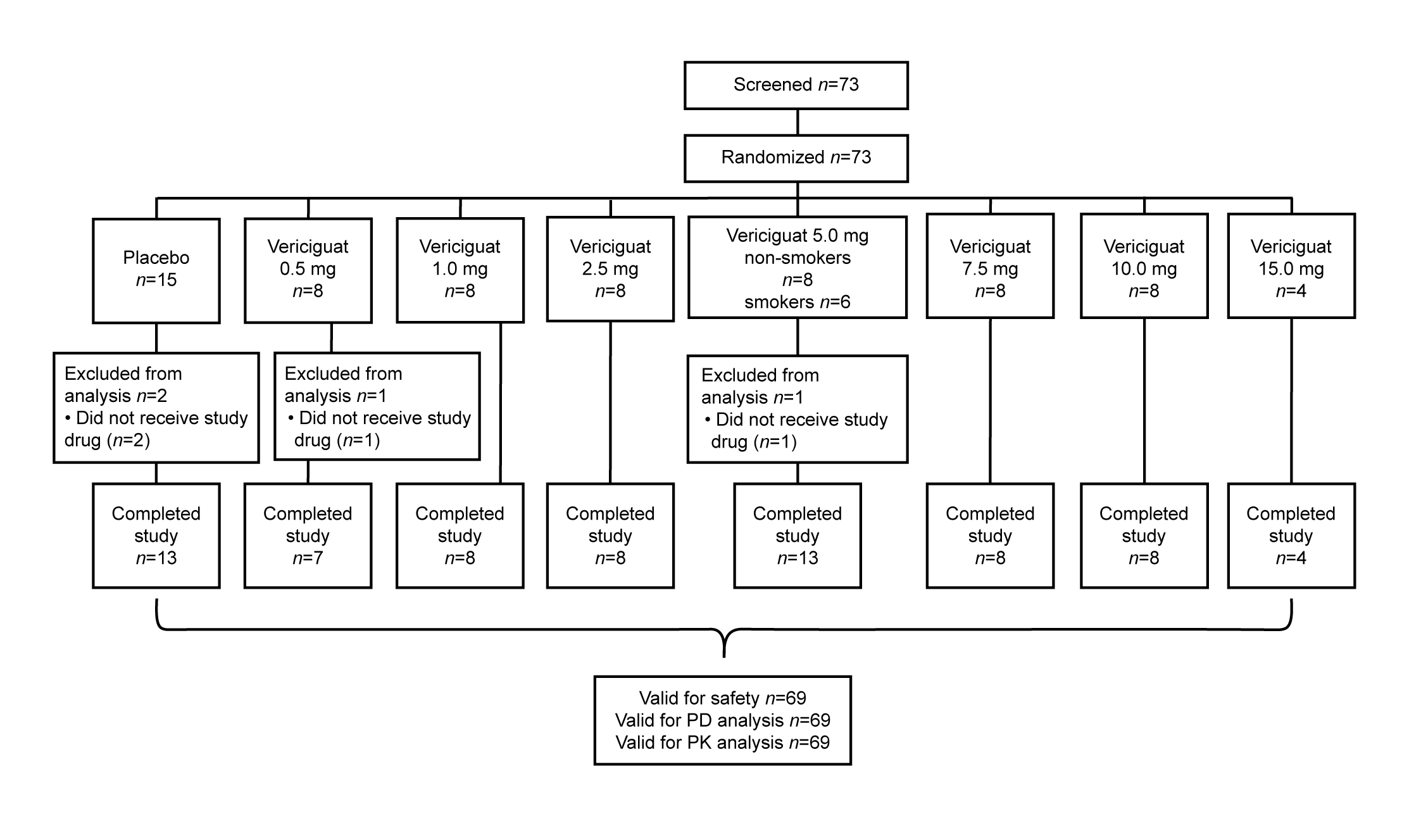

Supplement: Supplementary file 1 — (PNG 64 kb) [file 228_2020_3023_Fig3_ESM.png]

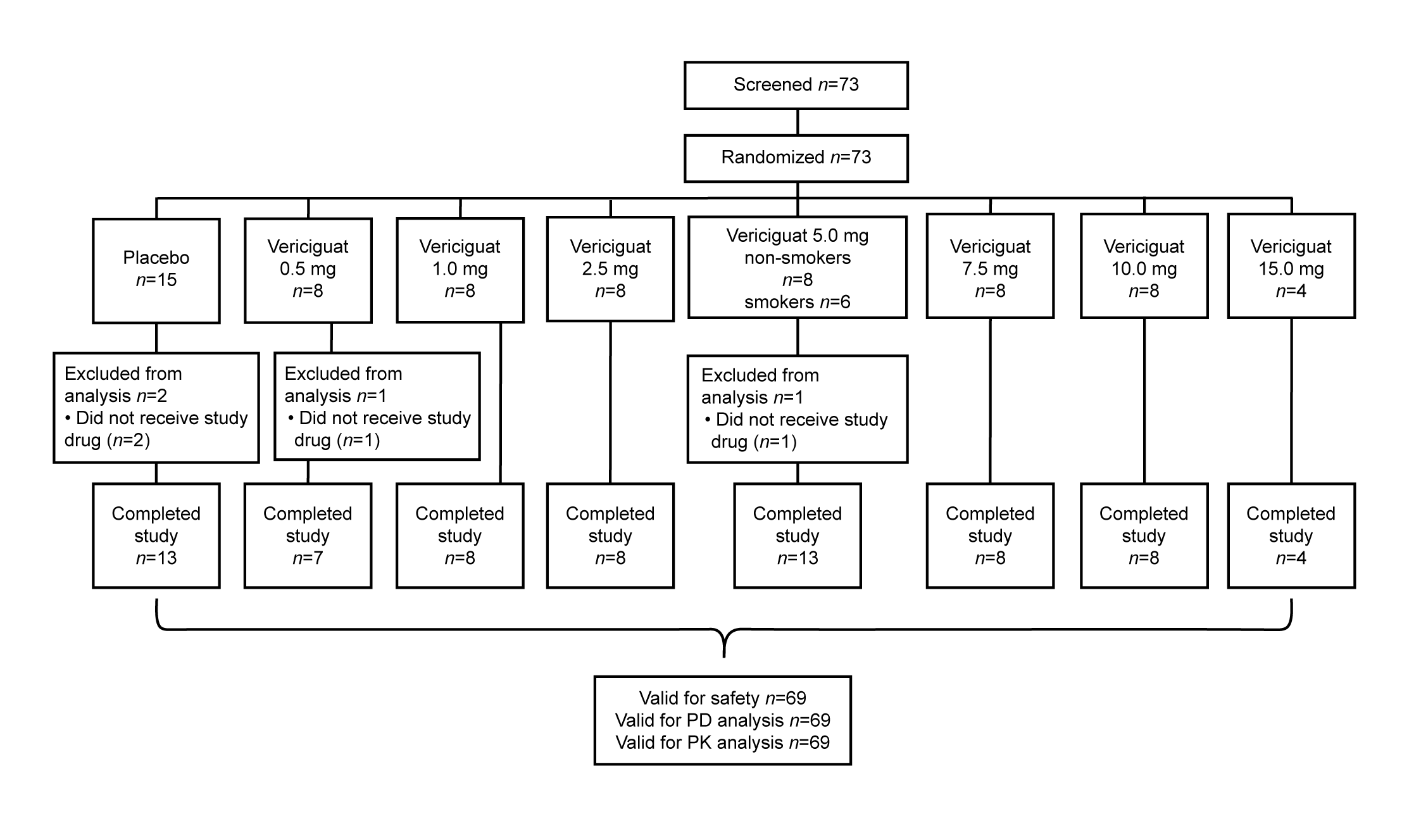

Supplement: Supplementary file 2 — High Resolution Image (TIF 371 kb) [file 228_2020_3023_MOESM1_ESM.tif]
