# Supplementary material for: Safety, pharmacodynamic, and pharmacokinetic characterization of vericiguat: results from six phase I studies in healthy subjects
Source: Eur J Clin Pharmacol. 2020 Oct 30;77(4):527–37. doi: 10.1007/s00228-020-03023-7 (PMC7935833; doi:10.1007/s00228-020-03023-7)
Supplement: Supplementary file 3 — (DOCX 76 kb) [file 228_2020_3023_MOESM2_ESM.docx]

Supplementary Appendix

Safety, pharmacodynamic, and pharmacokinetic characterization of vericiguat: results from six phase I studies in healthy subjects

Michael Boettcher^1^, Dirk Thomas^2^, Wolfgang Mueck^1^, Stephanie Loewen^3^, Erich Arens^1^*, Kenichi Yoshikawa^4^, Corina Becker^1^

^1^Clinical Pharmacology, Bayer AG, Wuppertal, Germany; ^2^Experimental Medicine, Bayer AG, Wuppertal, Germany; ^3^Chrestos Concept GmbH & Co. KG, Essen, Germany; ^4^Clinical Sciences, Research & Development Japan, Bayer Yakuhin, Ltd, Osaka, Japan
*Im Strasschen 12, D-40789 Monheim, Germany

**Supplementary Table 1** Pharmacokinetic parameters assessed

| **Pharmacokinetic Parameter** | **Abbreviation** |
| --- | --- |
| area under the plasma concentration versus time curve from zero to infinity for total (bound and unbound) drug after a single (first) dose | AUC |
| dose-normalized AUC calculated as AUC divided by dose per kg body weight | AUC_norm_ |
| AUC from time 0 to the last data point | AUC(0–t_last_) |
| dose‑normalized AUC(0–t_last_), calculated as AUC(0–t_last_) divided by dose per kg body weight | AUC[0–t_last_]_norm_ |
| percent AUC from the last data point to infinity | %AUC(t_last‑∞_); |
| maximum total (bound and unbound) drug concentration in plasma after SD administration | C_max_ |
| dose-normalized C_max_ (C_max_ divided by dose per kg body weight) | C_max,norm_ |
| time to reach C_max_ | t_max_ |
| half-life associated with the terminal slope | t_1/2_ |
| mean residence time | MRT |
| total body clearance of drug calculated after extravascular administration | CL/F |
| apparent volume of distribution during the terminal phase after extravascular administration | V_z_/F |
| amount excreted in the urine | AE_,ur_ |
| percent amount of drug excreted into urine | %A_E,ur_ |
| renal body clearance of drug | CL_R_ |
| points terminal |  |
| accumulation ratio calculated from AUC after multiple and SDs | R_A_AUC |
| accumulation ratio calculated from C_max_ after multiple and SDs | R_A_C_max_ |
| linearity factor of PK after multiple administration of identical doses calculated from AUC after multiple and SDs | R_LIN_ |

*PK,* pharmacokinetic; *SD*, single dose

Supplementary Table 2 Demographics of subjects in six clinical studies investigating the safety, pharmacodynamics and pharmacokinetics of vericiguat (safety analyses set)

|  | **Clinical study** | | | | | |
| --- | --- | --- | --- | --- | --- | --- |
| **Characteristic** | **SD1**  ***n*=69** | **SD2 *n*=36** | **MD1**  ***n*=48** | **MD2 *n*=43** | **MD3**  ***n*=46** | **BA1 *n*=16** |
| Male, n (%) | 69 (100) | 36 (100) | 48 (100) | 43 (100) | 46 (100) | 16 (100) |
| Ethnicity/Race (country) | European (Germany) | Chinese (Singapore) | Japanese (Japan) | European (Germany) | Chinese (China) | European (Germany) |
| Age, mean (s.d.), years | 34.9 (7.7) | 32.6 (6.8) | 27.1 (5.7) | 33.4 (7.4) | 30.8 (6.6) | 38.5 (5.5) |
| Weight, mean (s.d.), kg | 80.2 (10.2) | 68.6 (8.2) | 62.4 (9.1) | 81.8 (10.7) | 63.3 (7.0) | 82.6 (12.8) |
| BMI, mean (s.d.), kg m^-2^ | 24.5 (2.4) | 23.2 (2.1) | 21.2 (2.5) | 25.2 (2.5) | 22.5 (1.8) | 24.6 (3.1) |
| Smoker, n (%) |  |  |  |  |  |  |
| Current | 8 (12.0) | 8 (22.2) | 5 (10.4) | 8 (18.6) | 7 (15.2)^a^ | 2 (12.5) |
| Former | 19 (28.0) | 2 (5.6) | 16 (33.3) | 9 (20.9) | 8 (17.4)^a^ | 1 (6.3) |

^a^ *n*=45
*BA*, bioavailability; *BMI*, body mass index; *MD*, multiple dose; *s.d.*, standard deviation; *SD*, single dose

Supplementary Table 3 Subjects with TEAEs and listing of TEAES by preferred terms: SD1

|  |  | **Vericiguat dose (oral PEG solution)** | | | | | | |
| --- | --- | --- | --- | --- | --- | --- | --- | --- |
| **MedDRA Preferred term,** **n (%)** | **Placebo  (*n*=13)** | **0.5 mg  (*n*=7)** | **1.0 mg  (*n*=8)** | **2.5 mg  (*n*=8)** | **5.0 mg (*n*=13)** | **7.5 mg  (*n*=8)** | **10.0 mg (*n*=8)** | **15.0 mg  (*n*=4)** |
| Number of subjects with any AE | 3 (23.1) | 1 (14.3) | 2 (25.0) | 3 (37.5) | 8 (61.5) | 4 (50.0) | 5 (62.5) | 4 (100.0) |
| Sinus bradycardia | 0 | 0 | 0 | 0 | 0 | 0 | 0 | 1 (25.0) |
| Abnormal sensation in eye | 0 | 0 | 0 | 0 | 1 (7.7) | 0 | 0 | 0 |
| Ocular hyperemia | 0 | 0 | 0 | 0 | 2 (15.4) | 0 | 1 (12.5) | 0 |
| Abdominal discomfort | 0 | 0 | 0 | 0 | 0 | 1 (12.5) | 0 | 0 |
| Diarrhea | 0 | 0 | 1 (12.5) | 0 | 1 (7.7) | 1 (12.5) | 0 | 0 |
| Dry mouth | 0 | 0 | 0 | 0 | 0 | 0 | 0 | 2 (50.0) |
| Flatulence | 0 | 0 | 0 | 0 | 0 | 0 | 1 (12.5) | 0 |
| Nausea | 0 | 0 | 0 | 0 | 1 (7.7) | 0 | 1 (12.5) | 0 |
| Oral mucosal erythema | 1 (7.7) | 0 | 0 | 0 | 0 | 0 | 0 | 0 |
| Application site erythema | 0 | 1 (14.3) | 0 | 0 | 0 | 0 | 0 | 0 |
| Catheter site swelling | 1 (7.7) | 0 | 0 | 0 | 0 | 0 | 0 | 1 (25.0) |
| Feeling cold | 0 | 0 | 0 | 0 | 0 | 1 (12.5) | 0 | 0 |
| Feeling hot | 1 (7.7) | 0 | 0 | 0 | 0 | 0 | 0 | 0 |
| Vessel puncture site reaction | 1 (7.7) | 0 | 0 | 0 | 0 | 0 | 0 | 0 |
| Herpes virus infection | 1 (7.7) | 0 | 0 | 0 | 0 | 0 | 0 | 0 |
| Nasopharyngitis | 0 | 0 | 0 | 0 | 2 (15.4) | 0 | 0 | 0 |
| Scratch | 0 | 0 | 0 | 0 | 0 | 0 | 0 | 1 (25.0) |
| Subcutaneous hematoma | 0 | 0 | 0 | 0 | 0 | 0 | 1 (12.5) | 0 |
| C-reactive protein increased | 0 | 0 | 0 | 0 | 1 (7.7) | 0 | 0 | 0 |
| Lipase increased | 0 | 0 | 0 | 1 (12.5) | 0 | 0 | 0 | 0 |
| Limb discomfort | 1 (7.7) | 0 | 0 | 0 | 0 | 0 | 0 | 0 |
| Dizziness postural | 0 | 0 | 0 | 2 (25.0) | 0 | 0 | 1 (12.5) | 2 (50.0) |
| Headache | 0 | 0 | 1 (12.5) | 1 (12.5) | 2 (15.4) | 1 (12.5) | 0 | 2 (50.0) |
| Syncope | 0 | 0 | 0 | 0 | 0 | 0 | 0 | 1 (25.0) |
| Spontaneous penile erection | 0 | 0 | 0 | 0 | 1 (7.7) | 0 | 1 (12.5) | 0 |
| Nasal congestion | 0 | 0 | 0 | 0 | 0 | 1 (12.5) | 0 | 0 |
| Oropharyngeal pain | 0 | 0 | 0 | 0 | 1 (7.7) | 0 | 0 | 0 |
| Throat irritation | 0 | 0 | 0 | 0 | 1 (7.7) | 0 | 1 (12.5) | 0 |
| Hematoma | 0 | 0 | 1 (12.5) | 0 | 0 | 0 | 0 | 0 |
| Orthostatic hypotension | 0 | 0 | 0 | 0 | 0 | 0 | 0 | 1 (25.0) |

Subjects were counted once within each preferred term
*AE*, adverse event; *MedDRA*, Medical Dictionary for Regulatory Activities; *PEG*, polyethylene glycol; *SD1*, single-dose study 1; *TEAE*, treatment-emergent adverse event

Supplementary Table 4 Pharmacokinetic parameters of vericiguat in plasma following single oral doses of vericiguat 1.25–10.0 mg as IR tablet(s) in the fasted state (Day 1; study MD1)

| **Parameter, unit** | **Vericiguat dose (IR tablets)** | | | | | | | |
| --- | --- | --- | --- | --- | --- | --- | --- | --- |
|  | **1.25 mg (*n*=9)** | | **5.0 mg (*n*=9)** | | **7.5 mg (*n*=9)** | | **10.0 mg (*n*=9)** | |
|  | **Geometric mean  (range)** | **CV, %** | **Geometric mean (range)** | **CV, %** | **Geometric mean (range)** | **CV, %** | **Geometric mean (range)** | **CV, %** |
| AUC, µg•h L^-1^ | 1190  (958–1660) | 17.5 | 4140  (2900–5150) | 21.1 | 5140  (2660–7710) | 38.6 | 7410  (4840–11400) | 30.5 |
| AUC/D, h L^-1^ | 0.96  (0.77–1.33) | 17.5 | 0.83  (0.58–1.03) | 21.1 | 0.69  (0.35–1.03) | 38.6 | 0.74  (0.48–1.14) | 30.5 |
| AUC_norm_, kg•h L^-1^ | 60.7  (50.1–75.5) | 15.9 | 47.2  (36.9–64.2) | 19.0 | 45.1  (25.6–6.4) | 30.9 | 47.6  (29.8–64.8) | 29.9 |
| AUC_(0–24)_, µg•h L^-1^ | 703  (536–875) | 16.6 | 2540  (1780–3070) | 20.6 | 3230  (2110–4670) | 30.7 | 4410  (2870–6490) | 29.9 |
| AUC_(0–24)_/D, h L^-1^ | 0.56  (0.43–0.70) | 16.6 | 0.51  (0.36–0.61) | 20.6 | 0.431  (0.28–0.62) | 30.7 | 0.44  (0.29–0.65) | 29.9 |
| AUC_(0–24)norm_, kg•h L^-1^ | 35.8  (29.8–41.2) | 12.5 | 29.0  (22.5–36.3) | 16.0 | 28.4  (20.5–35.6) | 22.2 | 28.3  (19.1–36.8) | 27.7 |
| AUC(0–t_last_), µg•h L^-1^ | 1140  (891–1550) | 17.3 | 3960  (2810–4940) | 21.6 | 5010  (2650–7480) | 37.8 | 7190  (4750–11100) | 29.7 |
| AUC(0–t_last_)/D, h L^-1^ | 0.913  (0.713–1.241) | 17.3 | 0.792  (0.562–0.988) | 21.6 | 0.669  (0.354–0.998) | 37.8 | 0.719  (0.475–1.106) | 29.7 |
| AUC(0–t_last_)_norm_, kg•h L^-1^ | 58.0  (46.9–72.3) | 15.8 | 45.2  (35.8–62.0) | 19.4 | 44.0  (25.5–60.1) | 30.1 | 46.1  (29.4–62.7) | 29.0 |
| C_max_, µg L^-1^ | 62  (35–81) | 28.5 | 236  (159–315) | 28.9 | 324  (186–570) | 39.6 | 365  (228–580) | 37.1 |
| C_max_/D, L^-1^ | 0.0495  (0.0279–0.0647) | 28.5 | 0.0472  (0.0318–0.0629) | 28.9 | 0.0431  (0.0248–0.0760) | 39.6 | 0.0365  (0.0228–0.0580) | 37.1 |
| C_max, norm_, kg L^-1^ | 3.14  (1.94–4.24) | 22.8 | 2.69  (1.91–3.43) | 19.0 | 2.84  (1.75–4.31) | 28.2 | 2.34  (1.39–3.29) | 36.2 |
| t_max_^a^, h | 1.00  (0.75–3.00) | – | 1.00  (0.75–4.00) | – | 1.00  (0.50–2.50) | – | 2.50  (0.75–4.00) | – |
| t_1/2_, h | 22.3  (17.5–29.6) | 18.2 | 21.1  (12.5–33.7) | 31.0 | 17.9  (12.4–24.1) | 17.9 | 18.3  (15.1–25.7) | 20.1 |
| MRT, h | 28.3  (22.6–33.8) | 14.9 | 26.9  (19.0–39.1) | 21.3 | 24.0  (12.8–28.8) | 24.5 | 26.9  (21.4–32.7) | 13.8 |
| V_z_/F, L | 33.6  (26.7–55.6) | 25.4 | 36.8  (25.2–70.4) | 36.4 | 37.7  (24.9–56.1) | 29.9 | 35.7  (23.7–47.4) | 27.5 |
| CL/F, L h^-1^ | 1.05  (0.75–1.30) | 17.5 | 1.21  (0.97–1.73) | 21.1 | 1.46  (0.97–2.82) | 38.6 | 1.35  (0.87–2.07) | 30.5 |

^a^Median (range)
*AUC*, area under the plasma concentration versus time curve from zero to infinity after single (first) dose; *AUC/D*, AUC divided by dose (mg); *AUC_norm_*, AUC divided by dose per kg body weight; *AUC_(0–24)_*, AUC from time 0 to 24 hours after administration; *AUC_(0–24)_/D*, AUC_(0–24)_ divided by dose (mg); *AUC_(0–24)norm_*, AUC_(0–24)_ divided by dose per kg body weight; *AUC(0–t_last_)*, AUC from time 0 to the last data point; *AUC(0–t_last_)/D*, AUC(0–t_last_) divided by dose (mg); *AUC(0–t_last_)_norm_*, AUC(0–t_last_) divided by dose per kg body weight; *CL/F*, total body clearance of drug from plasma calculated after oral administration (apparent oral clearance); *C_max_*, maximum drug concentration in plasma after single dose administration; *C_max_/D*, C_max_ divided by dose (mg); *C_max,norm_*, C_max_ divided by dose per kg body weight; *CV*, coefficient of variation; *IR*, immediate release; *MD1*, multiple dose study 1; *MRT*, mean residence time; *t_max_*, time to reach maximum drug concentration in plasma after single (first) dose; *t_1/2_*, half-life associated with the terminal slope; *V_Z_/F*, apparent volume of distribution during terminal phase after oral administration

Supplementary Figure 1 CONSORT flow diagram. Patient disposition in the first-in-human study, SD1

*PD*, pharmacodynamic; *PK*, pharmacokinetic; *SD1*, single-dose study
